# Mothering a population: How Danish mothers experience newborn dried blood spot samples and their considerations about re-use of samples for research purposes

**DOI:** 10.18332/ejm/152322

**Published:** 2022-09-05

**Authors:** Francisca Nordfalk, Anja M. B. Jensen

**Affiliations:** 1 Center for Medical Science and Technology Studies, Section for Health Services Research, Department of Public Health, University of Copenhagen, Copenhagen, Denmark

**Keywords:** attitudes, experiences, newborn screening, research practices

## Abstract

**INTRODUCTION:**

Newborn dried blood spot (NDBS) samples have a primary clinical purpose of screening, but often also a secondary purpose of re-use for research purposes. This study investigates how Danish mothers experience the sample being taken, their considerations about potential re-use for research, and their reflections on the level of information they receive about NDBS samples and their re-use for research purposes.

**METHODS:**

This study is based on semi-structured interviews with 19 mothers of newborn babies, conducted within the first year after the birth. Mothers were recruited through online media and interviewed in their own homes or at the university. All interviews were coded thematically with an abductive approach.

**RESULTS:**

Generally, mothers have difficulties in recollecting the NDBS sample. Overall, they are pleased with the re-use of samples for research purposes. However, some mothers are concerned about the consent procedure. The mothers did not have one specific attitude towards more information about the research done using newborn samples. Based on our findings, we recommend a higher level of transparency regarding national genetic research in general; and, for the NDBS samples specifically, a more complete information and consent procedure. Further, we encourage more studies into what kinds of information parents might find useful about research based on NDBS samples.

**CONCLUSIONS:**

Mothers do not always remember experiencing the NDBS sample, are generally positive towards re-use for research purposes, but unsure about best information level.

## INTRODUCTION

Every year, millions of newborn babies participate in newborn screening programs worldwide^1^, one of them being newborn screening based on newborn dried blood spot (NDBS) samples. NDBS samples are filter paper blood samples. In Denmark, they are by standard taken 48–72 hours after birth, either by a midwife, a nurse specialized in postnatal care, or a medical laboratory technician. The samples are collected nationwide and most often in the hospital where the parents return for a visit, two to three days after the birth. The clinical purpose of NDBS samples is screening and in Denmark, newborns are screened for 18 different diseases, mainly related to metabolic disorders. After the initial screening, the samples are stored indefinitely at the Danish Neonatal Screening Biobank and can be re-used for a secondary purpose of research (regulated by Danish law, the Danish Neonatal Screening Biobank and the national committee on health research ethics)^2^. As such, the samples have a dual purpose: both identifying actual risks for the individual newborn baby and contributing to a larger research agenda that might potentially enhance the well-being of a population in the future. Based on interviews with Danish mothers of newborns, this article focuses on the intersection between clinical screening and re-use of the samples for research.

Newborn screening programs and the re-use of NDBS samples are often debated, especially in regard to ethical considerations^3-7^. Here, Denmark serves as a unique case, as a ‘data heaven’ and one of the most research radical countries^8,9^ where the interplay between wide-ranging access to medical data on Danish citizens, and its liberal legislation, make Denmark one of the most resource-rich countries for conducting health research. The NDBS samples are no exception. Since 1982, virtually all children born in Denmark have had the sample taken^2^. The NDBS sample thus provides a new genetic perspective to the story of Denmark as ‘the epidemiologist's dream’^10,11^. However, for all countries, the re-use of NDBS samples for research purposes is only feasible if the parents accept the sample being taken in the first place, and secondly if they also accept the NDBS samples being used for research.

Due to the principle of informed consent as determined by Danish Law, all parents are handed nationalized standardized written information about the sample when leaving the delivery room at the hospital. Earlier studies have shown, how parents generally do not receive further information about the sample or use for research purposes, when the sample is subsequently taken^12,13^. In Denmark, consenting to have the sample taken includes an embedded consent for having the sample stored and possibly used for research purposes. Therefore, we were interested in knowing whether parents were aware of the secondary research purpose before we talked to them about it. Earlier studies have shown how parents are not always knowledgeable about the newborn screening, or the possibility of refusing^14-16^, but in general support the use of NDBS samples for secondary research purposes^17-19^. Further studies have shown how parents prefer more information and only support research if they are informed and invited to give consent^14,20-24^. However, parents’ attitudes towards research participation have never been studied in a country of highly intensive research like Denmark. Furthermore, there seems to be a lack of attention to how families experience the NDBS samples at the moment they are taken. Our aims are: 1) to investigate how the mothers experience the NDBS samples being taken; 2) to explore mothers’ considerations as to the sample being re-used for research purposes; and 3) to gain knowledge about mothers’ reflections on the possibility of receiving more information about re-use of NDBS samples.

## METHODS

This study is based on semi-structured interviews with mothers of newborn babies. At the time of interviews, babies had an average age of 7 months. We recruited the participants through Facebook and two Danish online communities for parents, https://www.min-mave.dk and https://www.baby.dk. Facebook and online media have earlier been reported as being effective tools for recruitment in social science and health research^25,26^. Our post opened with ‘Dear friends with small children’ followed by an invitation to contact us if they wanted to be interviewed about their experiences with ‘having samples taken from their newborns after birth’. The post did not disclose our particular interest in the NDBS sample or the focus on re-use for research purposes, since we also wanted to explore whether mothers were aware of this. Our initial goal was 15 respondents based on the presumption of information redundancy^27^. Within 48 hours, the Facebook post had 60 comments, 23 people had shared it, and more than 25 people had expressed an interest to participate. Because of the Danish structures regarding parental leave where mothers most often take the majority of the leave, all who responded to the advertisement were mothers. When recruiting for interviews, we did not provide information about the use of NDBS samples for secondary purposes in order to avoid bias.

A question guide was developed and is given in the Supplementary file. Interviews were transcribed verbatim and coded thematically in accordance with our initial research questions in order to identify relevant themes, especially focused on the level of information among mothers and their overall attitude towards the re-use of samples^28,29^. In all, 50% of the interviews were checked for coding consistency and for overall thematic consistency. In this process, new themes appeared from the empirical material, such as how mothers experienced the particular moment of the taking of the sample, the wellbeing of the baby, relations to health professionals, trust, and hope. As such, our conceptual framework was devised according to the principles of abductive analysis^30^, committed to both deductive and inductive coding. All participants gave both oral and written consent. The participants have been anonymized and given a pseudonym for all representations including this article. The study meets all requirements of the Helsinki declaration, the GPDR and the Danish data protection agency. According to Danish regulations, this study does not require any ethics approval.

## RESULTS

### Participants and data material

A total of 19 mothers were interviewed, in 18 interviews (one interview was with two mothers) in the late summer of 2017. The mothers were aged 21–42 years, lived in the greater Copenhagen area and had at least a college/BA level of education; 17 mothers gave birth at the hospital, two had planned home births, and 11 were first time mothers. The participants chose the locations; 16 interviews took place in the homes of the mothers, two mothers preferred to meet at the university. The interviews lasted between 45 minutes and two hours, providing us with substantial insights into the experiences of mothers. When analyzing our 18 interviews, we obtained data saturation by reaching a point where we identified no new information or new themes^31^. Most dominant themes^28^ structuring the three following sections presented here were: (new) motherhood, wellbeing of baby, health professional guidance, research purposes, information level, participation, trust in welfare system, future hopes, and knowledge needs.

### How mothers of newborns experience having the NDBS sample taken

During our studies, we met Camilla, a first-time mother of a baby girl who after an otherwise unproblematic birth struggled with breastfeeding. The family was therefore invited to stay at the hospital for a few days after the birth. When Camilla was asked about the NDBS sample, she had some difficulties recollecting the details:

*‘Yes, I remember how ... Now, I have to dig a little. Well, they had to take; now I will start with what I remember first, it was right before we had to leave the hospital. We were there for two days. Then they took ... They checked her ears and then they took a little sample from her heel. Maybe, yes, I believe so. They took a small blood sample, maybe from the heel. Yes, because there was something about having to send it…’*

Because of Camilla’s extended hospital stay, she had the sample taken before they left the hospital, where the NDBS sample became part of a larger process. This perception was evident among many other parents in our study. They regarded the NBDS as part of a ‘bigger package’ making sure everything was fine with their baby, rather than it being a deliberate choice for them to participate in a national screening program leading to the creation of a population biobank of genetic material.

Some of the mothers did have a clear recollection about the particular moment the NDBS sample was taken from their newborn. Lisa, who had given birth to a healthy girl, Sally, just before her due date, went home after the birth, and then had to return to the hospital for the NDBS sample. She described her experience:

Lisa: *‘Then we are just told that Sally should be placed on that gurney. And the father, or one of us, can give her some sugar water. Then Robert starts giving her some sugar water and then the nurse starts pricking her in the heel, and Sally then starts screaming wildly …’*

Interviewer: ‘Did you and Robert ask any questions when the sample was taken?’

Lisa: *‘No, it was more a matter of practicalities, I think. We wanted to make sure Sally was in as little pain as possible. And how you were supposed to hold her. I imagined I was to hold her, but I wasn't ... Maybe I would've found it more obvious, just having given birth to a little baby, that it would be more comforting for the baby to be close to me. Rather than being put on a cold table, “There you go, you're going to be pricked.”’*

Lisa and Robert did not question the NDBS sample as such; Lisa was more interested in ensuring Sally was as comfortable as possible. The parental focus on the discomfort is not unnoticed in existing literature^12,13^, however, despite such recollections appearing regularly in our material, none of the mothers we interviewed ever considered the trauma sufficient to cause them to decline the sample’s being taken. Further, this suggests that what is most important for mothers is how they can take best care of their newborn when a needle has to be pricked in the heel, rather than what takes place with the actual blood sample. The well-being and care for the baby overshadows the complexity of the process of the NDBS sample.

A few of the mothers explained how they appreciated the time when the NDBS sampling took place, as this was their first opportunity to talk to a health professional after the birth. As Laura, the mother of August and Evelyn, told us about having the sample taken in their own home:

*‘… With August it was just really nice, when she (the midwife) came to our home. Because I got the sense that she also looked around to see what it is like here, and how safe it is for him to be here. And I liked that, really. Then we were allowed to be in our own bathroom and in the bed and it was very comforting, really. Especially at a time where many other parents were not able to go to the maternity hotel and have a safe start with some guidance. Therefore, it was really nice of her to come by here.’*

Concomitantly, as described more thoroughly elsewhere, the midwives and nurses would use the moments when NDBS samples were taken as a space to talk to and provide support for the parents. The NDBS sampling practices thus fulfil an additional role besides screening and research, providing a desired time slot to create the best possible start for mother and child, just as Laura experienced.

### Attitudes towards NDBS samples being used for research purposes

Individual experiences of the moment of sampling differ widely according to the hospital and whether or not the mother and baby are admitted after giving birth. However, the initial attitudes towards the NDBS samples were shared among many of our informants: they were pleased with the samples being used for research. It transpired that none of the eighteen mothers we interviewed knew before we told them. Upon revealing to them that the sample they had given, and almost forgotten, could be used for research purposes, the majority reacted like Barbara:

*‘Well, my immediate thought would be that it is just fine. Moreover, it is really good that you have the possibility of learning from samples that are already sort of lying there.’*

Their first reaction was to be pleased with the idea, tending to support the notion of ‘learning’ as Barbara stated, but interestingly, without needing to know the details or the purpose of that research. In addition, they considered the practice of re-using, and not wasting, a sample that had already been taken, to be reasonable. However, what really baffled the mothers was the consent procedure. When Lisa was informed about the use of samples for research, the following dialogue took place:

Lisa: *‘Well … Really, the first thought in my mind was, “Have they gotten permission to do that? Did I somehow indirectly agree to that, when I came with my child?” And we probably did ...’*

Interviewer: *‘Well, that is right. Practice today is that if you agree to have the sample taken, then you also consent to research and quality assurance tests and to storage in general.’*

Lisa: *‘… and really, I think it is fine to store them and possibly help research. I think it is just fine that you have some material to work with. And that you do not have to do it (the sample) again! Having something stored, so you do not have to bother more people.’*

Lisa wondered about permissions but believed it was important to help research. To her, the tangible practicality of re-using the sample ‘so you don't have to bother more people’, seems to neutralize her initial critical question regarding permission and her lacking recollection of whether she actually gave consent. This doubt was evident among many of the mothers in our study. Some thought they must have given consent for the sample to be used for research purposes. Others just questioned if this was really the practice. Among our interviewees, only one of the mothers seemed upset. Rose, who otherwise had a clear recollection of the sample and the setting, told us that she had made a specific choice about her sons’ biological material not being stored in another research trial in which they participated. When Rose was informed about the research on the NDBS sample, she said:

*‘This makes me rather outraged. Then I feel like it has no point that I opted out (of research) in one place when it is just automatically stored and can be used for research anyway.’*

Without the information that the sample is stored and possibly re-used for research purposes, mothers like Rose are deprived of control over the biological material of their children. Even if Rose was the only parent expressing this particular concern in our study, it is a significant point that needs to be taken seriously when discussing the transparency and legitimacy of handling and using samples like the NDBS samples.

In interviews, the mothers would also talk about their concerns in relation to this new knowledge provided to them. Barbara (as quoted above), while initially positive towards the samples being stored, also said to us:

*‘… I am also thinking that the legal policies can quickly change and then they will still have his sample. Then maybe in twenty years it might be legal to return with information that he has some genetic disposition or something specific. I would be uncomfortable with that. I would be insecure if that information could be used somehow. … Then as a final consequence, I know it is a bit paranoid to think, but as a final consequence, someone might sequence some of his genes. Then as a final, final consequence, if this ended up in the wrong place, it would be scary! Nevertheless, I am not so concerned that I would consider opting him out.’*

Some of the mothers did not express any worries, but those who did had rather similar views to Barbara. However, her words also express another view shared by all the mothers we interviewed: no matter their attitude or experience, none of them considered having their child’s NDBS sample removed from the biobank, which is possible in Denmark^32,33^. Even though some mothers such as Rose were critical of research with genetic material, or, like Barbara, feared the future possibilities and negative consequences of a stored sample, none of them perceived this research practice to be compromising in a way that would lead them to remove a sample from the biobank.

### Views on more information about research participation

When interviewing the mothers, we were also interested in knowing their attitudes towards possibly obtaining more information about the research conducted with both the NDBS samples and their child’s sample in particular. Mona, 31-year-old and first-time mother, articulated the contrasting views regarding need for more information:

*‘Dammit, that is really difficult! Because it is both good and bad. On one hand, you could be very overwhelmed if you participated in all sorts of research. Really, like information overload! On the other hand, there might also be something interesting, and some diseases I was not paying any attention to, where I could notice something that could benefit my own health. Then I would like to know. However, it is always difficult because there might also be … something that would be unsuitable to know.’*

Mona’s words encompass the complexity of many of the answers we received. There was no one standard attitude among the mothers we interviewed; the majority, like Mona, had conflicts about receiving more information. Initially, most thought it would be interesting to know. Laura said, taking her daughter Evelyn, as an example:

*‘I think it would be somewhat fun. I think I would have a positive mind-set like, “God, can our sample be used for that?” I think that would be my attitude, if they called and said, “We've found Evelyn's sample which we can use for a project, or we've already used it for this and that.” Then I think my reaction would be, “Really, how interesting! So cool, did you get something out of it?”’*

Laura did not consider it a threat to her or her children if the samples were used in research, and she contributed readily. She did express a wish for more information, but it did not seem to hold any great importance for her. Some of the mothers felt completely different, responding with content that not knowing would save them from a headache of questions. Another mother, Christina, said:

*‘I do not think I would like to know. I think it would uhm ... it would be too much for me. Unnecessary knowledge, which still would lead to all kinds of thoughts.’*

Even just knowing that the sample had been used could be stressful, without further information as to either research or result. Some parents would rather live in blissful ignorance, not knowing how and for what their children’s samples might be used in research.

We found many discrepancies both as to whether the mothers were even interested in more information and what kind of information might be useful for them. Given the fact that they were unaware that the samples are stored and can be re-used for research, supplying information about these research practices seems to get ahead of the information flow. Elizabeth, another the mother said:

*‘I think that with the current system, it needs to stay like this: it requires much more information, or some sort of security net, if you want to give that kind of feedback such a long time after and regarding something, you are not aware about being part of in the first place.’*

This suggests that if the Danish healthcare system continues with the current practice, where parents are not always aware that the NDBS samples are stored and possibly re-used, it would not make sense to start informing them about what kind of research is done with the samples.

## DISCUSSION

In a time with increasing demand for clinical samples to be available for research purposes, it is crucial to understand how those giving the sample experience and perceive these practices. From this study, we have learned how mothers have very different experiences of having an NDBS sample taken from their baby. Most parents have difficulties recollecting the details regarding the NDBS sampling, which illustrates that, with a newborn baby in your arms, NDBS samples are not highly prioritized. Some recall the discomfort of their newborn child and others remember the warmth of a visit by a midwife. Still, the majority would probably never have thought about this moment in time again, had we not asked. Likewise, the mothers expressed some uncertainty about the exact consent procedure. Still, it did not seem really to create any anger or distrust, unlike in earlier studies about NDBS samples, which indicate that parents were only positive towards re-use for research purposes if parental permission or consent were clear^20,21,34^. Our findings, regarding the mothers’ attitudes to the use of samples for research purposes, overall support earlier studies indicating that in general, mothers saw no problems in samples being used for research. It made sense to the mothers we spoke to, that a sample, which already existed, could be re-used for another purpose. Moreover, they generally found research to be a legitimate purpose for re-use. Another interesting result is that all mothers, even the few who expressed a critical attitude, said ‘Yes’ when we asked if they would do it again. Earlier studies have shown how parents in general would like more information about the NDBS samples^15,17,23^ and in some cases also about the re-use of NDBS samples for research purposes^24^. When considering the suggestion for more information about the research undertaken with the NDBS samples, we were therefore surprised to learn that a majority of the mothers did not really consider this valuable. In addition, when considering obtaining information about the specific re-use of their child’s sample, they were indecisive.

Based on the findings presented in this study, we recommend a higher level of transparency regarding national genetic research in general; and for the NDBS samples specifically, a more complete information process about the fact that the samples are being stored and can be used for research purposes. We believe this could improve current practice, ensure the legitimacy of the process, and help mothers find their way in the information, without risking the samples as a research resource. If we wish to sustain the NDBS samples as a research resource, we must attend to the experiences of the parents and the level of transparency regarding national research resources.

### Strengths and limitations

The overall strength of this study is our ethnographic approach. The interpersonal nature of our interviews, their length, and the fact that most of them were conducted in the homes of mothers, allowed for in-depth insights into the experiences of NDBS sample taking from a maternal perspective and an understanding of how the research participation of newborn babies interacts with care for and information to new mothers. A possible limitation to this finding is the fact that all the mothers who participated in our study had volunteered, thereby indicating that they already had a positive attitude towards research participation in general. However, we did talk to mothers who were otherwise critical of genetic research and the Danish health system. Still, they were not so worried about the possible re-use of their child’s NDBS sample so that they would consider opting out of research based on NDBS samples or removing the sample from the biobank. Another possible limitation to our qualitative study is the sample size, which provides limited opportunity for comparability to quantitative studies with similar focus. Future qualitative studies of this group of mothers could include a larger number of informants and checked more systematically for differences regarding, the age of the baby, the number of children, trust in the healthcare system based on previous experiences, and the importance of the relationship to the healthcare providers. In addition, our data is from the Danish context; a larger study focusing on cross-national experiences of mothers would be very interesting. Furthermore, in our study recall bias can be a limitation, mothers of babies might suffer from sleep deprivation affecting their memory, and often babies needed attention or milk from their mothers during interviews, all of which most likely interrupted the answers and the flow of conversation. In future studies, we recommend including fathers and to consider how or whether the critical circumstances of giving birth, the number of staff, or the hospital admission time, might affect parental experiences of NDBS.

## CONCLUSIONS

We have presented a range of experiences and attitudes that Danish mothers might have. Mothers often experience having difficulties in recollecting the newborn dried blood spot sample. Moreover, the mothers were overall comfortable with re-use for research purposes. Still, some mothers expressed concerns about the consent procedure. Our material showed a wide range of attitudes towards the level of information about the re-use of samples. If mothers are to be made more aware of the research value of the NDBS samples, we need to discuss further what information they need to receive and when to provide this information. We therefore recommend that future studies focus on what kind of information parents would find useful to make a sustainable research resource from NDBS samples.

## Data Availability

The data supporting this research cannot be made available for privacy reasons.
