# Supplementary material for: Mothering a population: How Danish mothers experience newborn dried blood spot samples and their considerations about re-use of samples for research purposes
Source: Eur J Midwifery. 2022 Sep 5;6:58. doi: 10.18332/ejm/152322 (PMC9442974; doi:10.18332/ejm/152322)
Supplement: Supplementary file 1 [file EJM-6-58-s1.pdf]

# Interview guide: Parents

Information before the interview:

- Thank you for your participation
- Short briefing about the project: policy, practice and experiences – I will primarily ask you about your experiences and your opinions. POLICYAID info paper.
- REMEMBER: You are the expert on your own experiences (there are no right or wrong answers) + there may occur things you have not thought about or can't remember – that's okay – we will just talk about it.
- Any questions so far?
- Data will be made anonymous
- If there are questions during the interview, you do not wish to answer, please just let me know.
- Ask about permission to record the interview – it is only for internal use and will be made anonymous everywhere
- Informed consent
- Let's begin ☺

ABOUT YOU:

- Who is the mother (father)? (Remember: born after 1982?) Education/work?
- Who is the child (remember name)? Birth – where and how?
- What motivates you to participate today with me in this interview?

EXPERIENCE:

*At first, I would like you to tell me about which tests were conducted and how you experience them.*

- Which tests do you remember?
- Where were you when the test(s) ware taken and who took the test(s)?
- What happened before and after the test(s) were taken?
- Did you talk about anything else at the situation where the test(s) were taken?
- You had recently become parents – how did you feel the day the test(s) were taken?

(Repeat with the NDBS samples, if they do not mention it themselves – that is my case)

[in Danish "PKU-test" for PHENYLKETONURIA]

*I have chosen the NDBS samples as my case, therefore I would like to know more about how you experienced the sample and what kind of information you received about the NDBS samples?*

- Do you remember situations where you received information about the NDBS samples – when?
- Do you remember how the purpose of the sample was explained to you?

- Do you remember something that was crucial/important for you to know?
- Did or do you experience missing any information? (And have you tried to find answers?)
- Did you have any questions while the sample was taken?
- Did you consider declining the sample? → What would have happened if you had declined?

RESEARCH:

*I would like to know more about your position on the NDBS samples and its use.*

- What do you think happens to the sample after it is taken?
- Is there something you do not want them to use your child's sample for?

*As you may have been told or read about in the information material, after the initial screening of the test, it is stored in a biobank and at the State Serum Institut. Therefore, if the child needs the sample, they have it stored. Furthermore, they can use the sample for quality assurance and research.*

- Did you know this before I told you?
- Do you remember if you talked about it? Or did you consider *if* the sample was being stored?

*As mentioned, the sample can be removed from the biobank to be used for research. They (researchers) will often look for genes that can explain diseases.*

- What are your thoughts on NDBS-samples being used for research?
- If it was possible, would you have chosen that the sample should not be used for research? (The three circles)
- Are there any research based on NDBS-sample that you would feel uneasy or anxious about? Why/ why not?
- Or *someone* you would rather not see have access to the information in your NDBS sample? Why / why not? (private /public /public administrative → motive/interest)
- Do you know what to do if you no longer want yours or your child's NDBS-sample to be used in research? What is your immediate thought?
- Would you like to know more about what research are using the NDBS samples? → How do you think this information should be provided to you? → Do you check those places? [Triangulation - eg sundhed.dk]

DATA COLLECTION IN GENERAL:

*We have talked about the NDBS-sample and how it can be utilized. But you also participate in health-related research in other ways. I would like to ask you about this here at the end…*

- Have you seen stories in the news about "this many Danes have this and that (disease)?" or "Danish studies reveal that this and that causes a risk for cancer"? Do you ever think about that these

studies may draw on your data? Have you ever talked about this with someone or heart anyone talk about it?

- Do you think there is a difference between if the data is blood samples with DNA or health information?
    - REGISTER
    - JOURNAL FROM DOCTOR
- What is important or relevant for you regarding participating in health related research in what is considered an *anonymous* way?
- Are there something(s) you do not think the information in your patient journal should be used for? Or someone the information should not be shared with? What are your considerations?
    - PUBLIC vs. PRIVATE
    - PUBLIC ADMINISTRATION and PUBLIC SOCIAL SUPPORT
- Does it make a difference for you, if the information can be traced back to you (anonymization, pseudonymisation)? Which difference?
    - What would be the consequence if it was not anonymous? (Who would use that information?)
- Does it make a difference to you to be asked for permission for your information to be used?
    - In practice, how could that work?
    - How do you think, for instance, side effects of treatments should be investigated?
    - In your opinion, whose data is it really? Who should decide on the use?
- Does the fact that others can use your health information influence what you chose to tell your doctor and the way you tell it?
- What does it mean for you to be able to participate and contribute to research?
- How do you think the results of Danish research can influence you?

CONCLUSIONs:

- Do you have any experiences or comments on research participation that we did not get into? Or any questions?
- May I contact you again?
- Thank you 😊

© 2022 Nordfalk F. and Jensen A. M. B.
